# Co-Assembled Nanosystems Exhibiting Intrinsic Fluorescence by Complexation of Amino Terpolymer and Its Quaternized Analog with Aggregation-Induced Emission (AIE) Dye

**DOI:** 10.3390/nano14201631

**Published:** 2024-10-11

**Authors:** Michaila Akathi Pantelaiou, Dimitrios Vagenas, Evangelos S. Karvelis, Georgios Rotas, Stergios Pispas

**Affiliations:** 1 Theoretical and Physical Chemistry Institute, National Hellenic Research Foundation, 48 Vassileos Constantinou Ave., 11635 Athens, Greece; akathi39@gmail.com (M.A.P.); dimitrisv98@gmail.com (D.V.); 2 Section of Organic Chemistry and Biochemistry, Department of Chemistry, University of Ioannina, 45110 Ioannina, Greece; ch05879@uoi.gr (E.S.K.); rotasgiorgos@uoi.gr (G.R.)

**Keywords:** aggregation-induced emission, enhanced fluorescence, polymeric nanomaterials, bioimaging

## Abstract

Aggregation-induced emission dyes (AIEs) have gained significant interest due to their unique optical properties. Upon aggregation, AIEs can exhibit remarkable fluorescence enhancement. These systems are ideal candidates for applications in bioimaging, such as image-guided drug delivery or surgery. Encapsulation of AIEs in polymeric nanocarriers can result in biocompatible and efficient nanosystems. Herein, we report the fabrication of novel nanoaggregates formulated by amino terpolymer and tetraphenylethylene (TPE) AIE in aqueous media. Poly(di(ethylene glycol) methyl ether methacrylate-co-2-(dimethylamino)ethylmethacrylate-co-oligoethylene glycol methyl ether methacrylate), P(DEGMA-co-DMAEMA-co-OEGMA) hydrophilic terpolymer was utilized for the complexation of the sodium tetraphenylethylene 4,4′,4″,4‴-tetrasulfonate AIE dye. Fluorescence spectroscopy, physicochemical studies, and self-assembly in aqueous and fetal bovine serum media were carried out. The finely dispersed nanoparticles exhibited enhanced fluorescence compared to the pure dye. To investigate the role of tertiary amino groups in the aggregation phenomenon, the polymer was quaternized, and quaternized polymer nanocarriers were fabricated. The increase in fluorescence intensity indicated stronger interaction between the cationic polymer analog and the dye. A stronger interaction between the nanoparticles and fetal bovine serum was observed in the case of the quaternized polymer. Thus, P(DEGMA-co-DMAEMA-co-OEGMA) formulations are better candidates for bioimaging applications than the quaternized ones, presenting both aggregation-induced emission and less interaction with fetal bovine serum.

## 1. Introduction

Numerous advancements in the field of photophysics have been made in the last few years. A newly discovered fluorescent phenomenon is aggregation-induced emission [1]. AIE was first introduced by Tang et al. in 2001 [2]. The group observed that, upon aggregation of 1-methyl-1,2,3,4,5-pentaphenylsilole in solution, the emission was boosted. A major problem for many fluorophores is the quenching of their emission in the aggregation state due to interactions, like π-π stacking [3]. On the contrary, in AIEgens, aggregation leads to an enhancement of luminescence [4]. Many mechanisms for the phenomenon were proposed, such as J-aggregation, E/Z isomerization, conformational planarization, and others. Tang et al. suggested the restriction of intramolecular motion (RIM) mechanism, which is generally accepted as the main mechanism of AIE [5,6]. RIM mechanism in AIEgen solution presents weak or no emission, but interestingly, in aggregates or in the solid state, the emission is boosted [7].

As AIEgens have become an important class of materials, research in this field has flourished, and many new materials have been discovered. The first fluorogens presented rotor moieties such as tetraphenylsilole (TPS) and TPE. In the following years, researchers utilized them as building blocks for the creation of new structures or the alteration of ACQ molecules to AIEgens [8]. Other major AIEgens include 1-[4-(1,2,2-triphenylvinyl)phenyl]pyrene (TPEPy), 4,7-Bis [4-(1,2,2-triphenylvinyl)phenyl]benzo-2,1,3-thiadiazole (BTPETD), tetraphenylpyrazine (TPP), thiophenes, and 9,10-distrylanthracene (DSA) derivatives [9]. Natural substances such as berberine have also been reported in the literature [10]. Among AIEgens, TPE and its derivatives have shown the most promising research results for biomedical applications. Sulphonated TPE can result in water-soluble systems with a negative outer charge that can target and interact with many molecules [11].

Polymeric nanocarriers are an important class of nanomaterials synthesized from natural or synthetic sources. The vast diversity of monomers and polymerization conditions can result in numerous different nanoparticles. This can lead to differences in size, shape, charge, and functionalization of the surface of the nanomaterial [12]. Such properties can result in the utilization of polymeric nanocarriers in nanomedicine. By tuning these properties, the nanomaterials can have reduced toxicity or better stability and biocompatibility. These systems are ideal candidates for drug delivery applications in neurodegenerative diseases, cancer, gene delivery, or imaging [13,14].

Polymer–dye hybrid nanosystems are a novel class of nanocarriers. These systems can present the AIE phenomenon. Luminophores can be encapsulated and aggregated in the core of nanoparticles. Another method is the incorporation of luminophores in polymeric chains that can then self-assemble into micelles or other nanostructures. These systems can present reduced toxicity, better biocompatibility, and thus act as delivery systems. Hu et al. loaded a TPE–TPA–DCM dye in a triblock copolymer for siRNA-mediated gene therapy [15]. In another study, the drug fluorouracil-1-acetic acid was arched to the TPE core and then encapsulated in a polymeric nanoassembly [16]. Electrostatic supramolecular assembly can also result in a loaded polymeric nanocarrier. In a recent study, the authors formed a nanoparticle of poly(allylaminehydrochloride) (PAH) and a TPE derivative, tetra-anionic Su-TPE, as an AIE probe [17]. A polymer with AIE features was synthesized by Li et al. Specifically, a TPE derivative was linked with hyaluronic acid and self-assembled in micelles for the delivery of paclitaxel [18]. The same strategy was followed by She et al. who incorporated TPE in modified chitosan and its quaternized form [19,20]. Finally, a hyperbranched polymeric core–shell nanoparticle was synthesized by the incorporation of TPE in polyglycidol [21].

Applications of AIEgens are vast spanning various fields from optoelectronics (OLEDS, sensing) to nanomedicine (bioprobes, image-guided drug delivery) [3]. A major advancement has been made in the field of bioimaging. First of all, with the help of AIE, microscopic imaging can be achieved [22]. AIE probes are photostable and present high sensitivity. Another important application is image-guided drug delivery, where AIEs can help researchers locate the route of the drug to a specific cell or tissue. Image-guided surgery can also benefit from these molecules, as it is of utmost importance to have a highly emissive molecule during the surgery procedure [23]. In cancer treatment, AIEgens can help with better and more efficient image-guided phototherapy [24].

Reversible addition fragmentation chain transfer (RAFT) polymerization is considered one of the most important polymerization methods in polymer science. This polymerization technique was first reported by CSIRO in 1998 [25]. RAFT is a controlled polymerization technique with precise control over the final synthesized polymer products. It is considered an important technique for the production of multiblock copolymers with complex architectures (block, star, and hyperbranched). The general polymerization mechanism consists of the stages of initiation, chain propagation, and termination [26]. RAFT techniques can be utilized for the easy polymerization of a vast range of monomers in many reaction media and polymerization conditions [27]. Fine tuning of the characteristics of polymers is of great importance for applications in nanomedicine and optoelectronics, making RAFT one of the most important techniques for the synthesis of materials for applications in these areas [28,29].

In this study, we aim to formulate co-assembled polymer–AIE dye nanosystems and study their physicochemical and photophysical properties as well as the aqueous solution behavior as a function of polymer charge density, polymer/dye weight ratio, and solution ionic strength in the presence of serum proteins. Sodium tetraphenylethylene 4,4′,4″,4‴-tetrasulfonate dye was encapsulated by an amino polymer and its quaternized analog. The P(DEGMA-co-DMAEMA-co-OEGMA) statistical terpolymer, which was previously synthesized by our group, was utilized for the formulation of polymeric nanocarriers [30]. Firstly, the physicochemical characterization of the dye took place by ultraviolet–visible spectroscopy (UV-Vis), fluorescence spectroscopy (FS), and dynamic light scattering (DLS) in order to understand the behavior of the dye in an aqueous solution. The co-assembly of the dye and the polymer took place in three different concentrations of the dye in an aqueous solution. All systems were characterized by UV-Vis, FS, DLS, and electrophoretic light scattering (ELS). The behavior of the particles was also investigated in the FBS/PBS solution and in the presence of salt. The measurements indicated the successful fabrication of nanosystems exhibiting enhancement in emission. In order to investigate the phenomenon of electrostatic complexation, the polymer was quaternized. The quaternization was confirmed by nuclear magnetic resonance (NMR) measurements. Following that, the complexation was repeated, and the experiments indicated a stronger emission. Overall, the aim of this study is to formulate polymer nanocarriers loaded with an AIE dye. Encapsulation of AIEgenes in polymeric nanocarriers can result in more biocompatible and efficient nanosystems compared to pure dye. Also, the dye aggregation phenomenon is studied in the presence of the oppositely charged copolymers, together with the photophysics of the mixed systems and the interaction with serum proteins as a first step to their biocompatibility assessment.

## 2. Materials and Methods

### 2.1. Materials

In this study, the in-house synthesized P(DEGMA-co-DMAEMA-co-OEGMA) terpolymer (M_w_ = 19,500, Đ = 1.25, weight ratio of monomers 43:44:13) was utilized for the formulation of nanocarriers [30]. Tetrahydrofuran (THF), iodomethane (CH_3_I), and *N*,*N*-dimethylformamide-d_7_ were purchased from Sigma Aldrich (Athens, Greece).

### 2.2. Quaternization of P(DEGMA-co-DMAEMA-co-OEGMA)

For the quaternization reaction, 200 mg of the polymer was placed in a round flask, 10 mL of THF was added, and the mixture remained under mild stirring. After the complete polymer dissolution, 52 μL of CH_3_I was added. The flask was covered with aluminum foil and sealed with a rubber septum. The reaction was left for 24 h under stirring at room temperature. Then, the solution was transferred into a vial. The vial was put in a water bath, and the solution was stirred until solvent evaporation. Afterwards, the quaternized polymer was placed in the dynamic vacuum for 24 h for drying.

### 2.3. Co-Assembly of Dye-Loaded Polymeric Nanocarriers

The co-assembly of the two polymers and the dye was carried out in deionized water. First, the calculated amounts of the polymers were dissolved under mild stirring for 30 min. Solutions with c = 10^−3^ g/mL and at pH = 7 were prepared. Then, for each polymer, the proper amount of dye was calculated and added to 0.5 mL of water. The solutions were left for 10 min on the bench. After that, three different ratios of polymer/dye, i.e., 1:0.25, 1:0.5, and 1:1 *w*/*w*, were prepared. The proper amount of dye solution was transferred to 5 ml of each polymeric solution under stirring for 3 min. Solutions of the neat polymers were also kept as reference. Finally, a pure solution of dye was prepared with a dye concentration similar to that of a 1:0.5 ratio.

### 2.4. Effect of FBS and Ionic Strength

The effect of fetal bovine serum protein solution on the formed nanoparticles was studied by the mixing of the FBS/PBS (1:9 *v*/*v*) solution with each nanoparticle sample. Specifically, 150 μL of each sample was added in 1.5 mL of the FBS/PBS solution, left for one hour, and then measured in the DLS instrument. In order to study the effect of salt on the formed polymer–dye nanoparticles, 850 μL of each nanoparticle solution was added to 150 μL of 1M NaCl and measured after 1 h by DLS.

### 2.5. Characterization Methods

#### 2.5.1. Proton Nuclear Magnetic Resonance Spectroscopy (^1^H-NMR)

The extent of the quaternization reaction was determined by ^1^H NMR. A Varian 300 (300 MHz) spectrometer equipped with the Vjnmr software (OpenVnmrJ 1.1A) was used for the measurement. The solvent used was deuterated DMF in a final polymer solution concentration c = 1 mg/mL. The spectra analysis was performed by using the MestReNova software v. 6.0.2 by MestReLabs (Santiago de Compostela, Spain).

#### 2.5.2. Dynamic Light Scattering (DLS)

DLS measurements were performed on an ALV/GS-3 compact goniometer system (ALV GmbH, Hessen, Germany). The laser source was a JDS Uniphase 22mW He-Ne operating at a 632.8 nm wavelength (JDS, Milpitas, CA, USA). An ALV/LSE-5003 scattering unit was utilized as the electronic interface system including a stepper motor guide and limit switch control, while the ALV-5000/EPP with 288 channels was used as the multi-τ correlator. Both autocorrelation functions and the intensity were taken as an average of five measurements. The measuring angle was set at 90°. For the analysis, the CONTIN algorithm was used. Before the experiments, the samples were filtered with 0.45 μm PVDF filters.

#### 2.5.3. Electrophoretic Light Scattering (ELS)

The surface charge of nanocarriers was determined through ELS. The Nano Zeta Sizer instrument (Malvern Panalytical, Malvern, UK) from Malvern was utilized. The instrument was equipped with a4 mW He–Ne laser, operating at a 633 nm wavelength, and measurements were taken at a scattering angle of 173°. For the analysis, the Smoluchowski equation was used. Each sample was scanned 20 times, and the average value was determined.

#### 2.5.4. Fluorescence Spectroscopy (FS)

The determination of aggregation-induced emission was carried out by fluorescence spectroscopy measurements. All solutions were diluted with deionized water at a ratio of 1:10. Spectra were recorded with a NanoLog Fluorometer (Horiba Jobin Yvon, Kyoto, Japan). A NanoLED440 nm laser with a pulse width of 100 ps was the excitation source. The detector used was a UV TBX-PMT (250–850 nm) by Horiba Jobin Yvon.

#### 2.5.5. Absorption Spectroscopy (UV-Vis)

Absorption spectra of samples were recorded in a Perkin–Elmer (Lambda 19, Waltham, MA, USA) UV-Vis-NIR spectrophotometer. All solutions were diluted in deionized water at a ratio of 1:10 and placed in quartz cells for measurements.

## 3. Results and Discussion

### 3.1. P(DEGMA-co-DMAEMA-co-OEGMA)–Dye Interactions

#### 3.1.1. P(DEGMA-co-DMAEMA-co-OEGMA) Synthesis

For this study, the P(DEGMA-co-DMAEMA-co-OEGMA) polymer that was previously synthesized in-house was utilized. The synthesis was carried out by RAFT polymerization. This specific polymer was chosen due to its stimuli-responsive properties as an ideal candidate for AIE applications. As proven by previous studies, it is an amphiphilic polymer that can self-assemble and create polymeric micelles in an aqueous medium. Furthermore, PDEGMA has been proven to be a biocompatible homopolymer, which can be utilized for biological applications. PDMAEMA contains a tertiary amine group that can be used for the electrostatic complexation of negatively charged moieties. As the dye utilized for this study was negatively charged, the complexation could be a result of the negative charge of the dye interacting with the positive charge of the DMAEMA segments [30].

#### 3.1.2. Synthesis and Characterization of Sodium Tetraphenylethylene 4,4′,4″,4‴-Tetrasulfonate Dye (BK11)

The synthetic procedure for the dye (BK11) is described in detail in the Supplementary Materials Section. Identification of the chemical structure was carried out by NMR (Figures S1–S3). As polymeric nanocarriers need to be used in biological applications, water was chosen as the solvent for all the experiments. Due to this, the dissolution of the dye was carried out in an aqueous medium in order to study its photophysical properties. UV-Vis measurements resulted in two characteristic absorption peaks at 290 nm and 317 nm (Figure S4). Before conducting fluorescence experiments and in order to qualitatively investigate the fluorescence of the solution by the naked eye, the dye solution was placed under a UV lamp at 365 nm (Figure S6). Fluorescence measurements were performed at two different excitation wavelengths. The 317 nm wavelength was selected due to absorbance data, while the 365 nm is preferred for experiments concerning biological applications. As it is proven in Figure S5, the dye presents fluorescence for both excitation wavelengths. For the excitation wavelength at 317 nm, the maximum peak is observed at 491 nm, while for the excitation wavelength of 365 nm, the maximum peak is observed at 480 nm (Tables S1 and S2) [31]. DLS measurements were carried out for the determination of the possible aggregation of the dye in water. In Figure S7 and Table S3, it is shown that the dye alone can present aggregation with the formation of particles with a size of 96 nm. These particles can explain the fluorescence emission that was measured.

#### 3.1.3. Co-Assembly of P(DEGMA-co-DMAEMA-co-OEGMA) and BK11 Dye

**Scheme 1:**
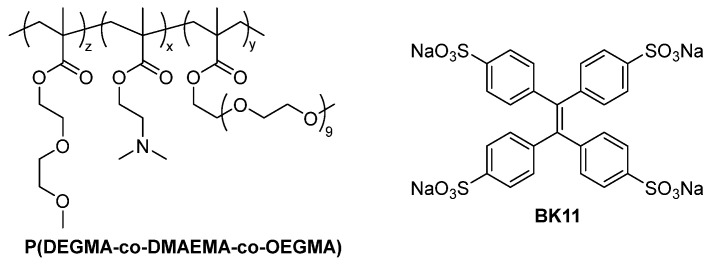
Structures of P(DEGMA-co-DMAEMA-co-OEGMA) copolymer and BK11 dye.

**Figure 1:**
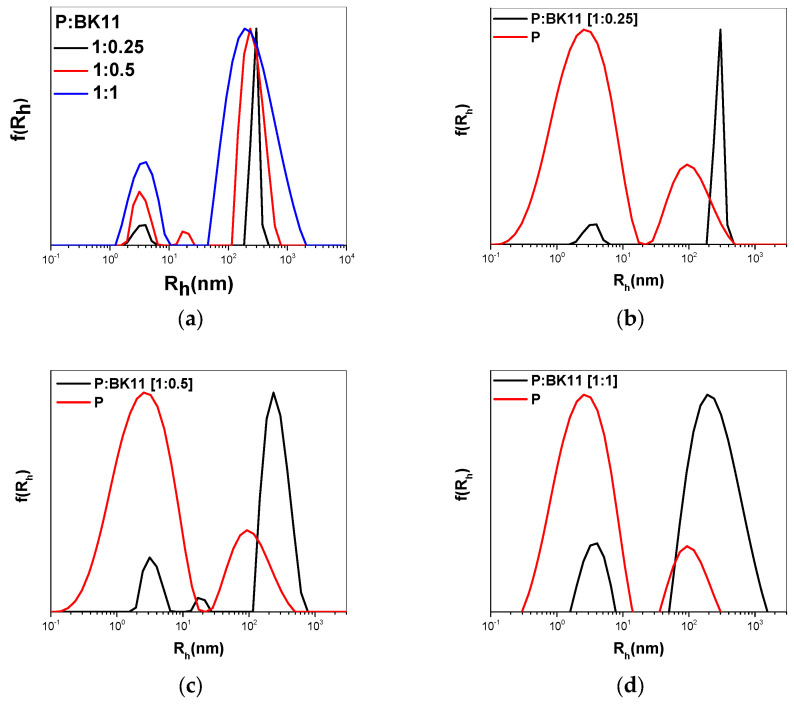
DLS measurements: (**a**) polymeric nanocarriers with loading ratios of 1:0.25 (black), 1:0.5 (red), and 1:1 (blue); (**b**) DLS measurements of polymer (red) and polymer–dye at ratio of 1:0.25 (black); (**c**) DLS measurements of polymer (red) and polymer–dye at ratio of 1:0.5 (black); and (**d**) DLS measurements of polymer (red) and polymer–dye at ratio of 1:1 (black).

The co-assembly of polymer–dye was carried out in three different loading ratios (by weight, *w*/*w*, Scheme 1). In order to observe the effect of the dye concentration on the system, DLS and ELS measurements were conducted and are presented in Table 1. As the pure polymer is amphiphilic, self-assembly is expected in a water medium. Plain polymeric nanoparticles were measured with a size of R_h_ = 103 nm, exhibiting a low scattering intensity of 23 Kcps. A peak at 2.3 nm depicts the presence of free polymeric chains in the medium. The co-assembly of polymer–dye in the formulations leads to particle sizes varying in the range of 245–283 nm (Figure 1a–d). Smaller particle sizes can be attributed to free polymeric chains. Also, zeta potential measurements revealed some non-monotonic variations in the negative charge of the particles as the negatively charged dye loading into the carriers was increased, with the most negative aggregates observed at the ratio of 1:0.25. In this case, the polymer carries tertiary amine groups, which are not all fully protonated. Co-assembled nanoparticles arising from each formulation can present a different and special internal structure and arrangements of the components where the negative charge of the dye can be in the interior of the nanoassembly or not. In the case of the 1:0.5 ratio, we believe that the charges are mostly residing in the interior of the nanoparticles due to the specific stoichiometry and the adapted polymer chain conformations within the mixed aggregates.

**Figure 2:**
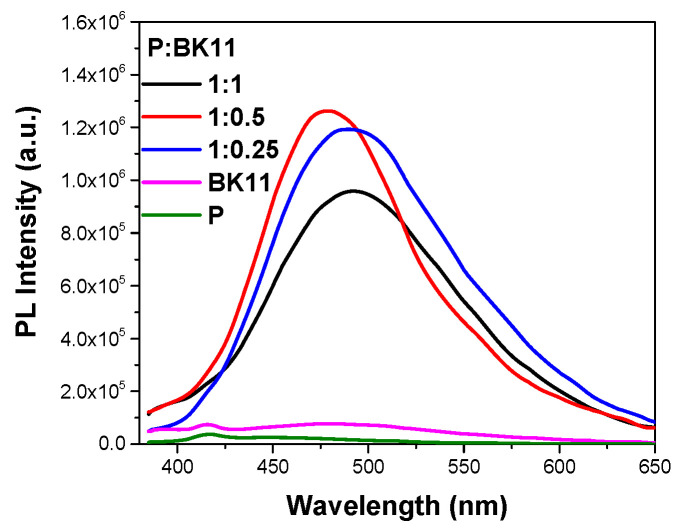
FS measurements conducted on polymer (green), dye (purple), and P:BK11co-assembled nanosystems with loading ratios of 1:0.25 (blue), 1:0.5 (red), and 1:1 (black) one day after preparation (spectra shown are not normalized to the moles of dye present in solution).

The polydispersity index is higher in the case of sample 1:1, and as Figure 1a depicts, the peaks are broader. Figure 1a–d show the presence of co-assembly and the complexation of the dye in the nanocarriers. In all cases, the plain polymer solutions present a higher intensity in the peak of 2.3 nm. After the addition of the dye, all samples present a change in the intensity of the peaks. Peaks attributed to polymer chains are lower, while peaks of larger nanoparticles are higher, indicating the incorporation of polymeric chains into co-assembled nanoparticles. Fluorescence experiments were carried out in order to prove the AIE phenomenon. In TPE, the negatively charged sulfonated groups interact electrostatically with the positively charged polymer. The rotation of the dye rings is restricted, thus giving rise to the AIE phenomenon via the restriction of intramolecular rotation (RIR) mechanism [6]. The excitation wavelength was selected to be 365 nm as it is suitable for biological applications. As depicted in Figure 2, the plain dye presents a near-zero intensity emission. In the case of polymeric co-assembled nanocarriers, an increased fluorescence is observed for all polymer–dye ratios (Table S4). The highest intensity is presented in formulation 1:0.5 at 479 nm. Furthermore, in ratios 1:1 and 1:0.25, a redshift is observed [32]. In each nanoparticle formulation, the loading ratio plays a crucial role in the final structure of the particles, while nanocarriers may reorganize after some time, which will affect the aggregation state of the dye molecules, resulting in the redshifts of the FS spectra. In the case of UV-Vis measurements, as the dye concentration increased, an increase in the absorbance was observed (Figure S8).

**Figure 3:**
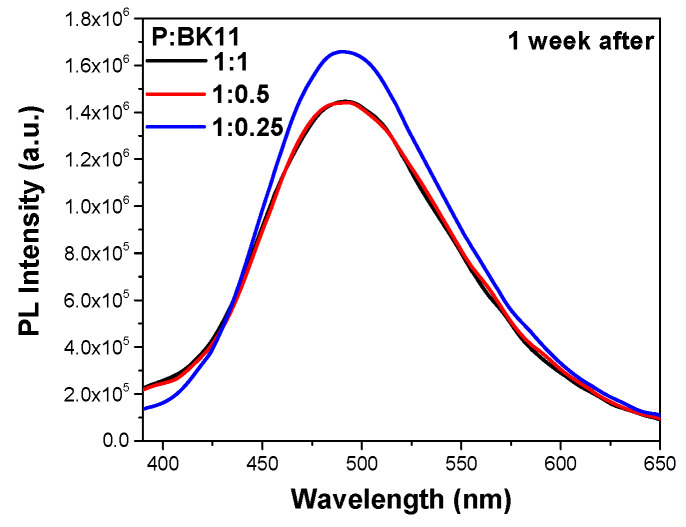
FS measurements of P:BK11 nanosystems with loading ratios 1:0.25 (blue), 1:0.5 (red), and 1:1 (black) conducted one week after preparation.

FS measurements were repeated one week after the preparation of formulations in order to investigate the stability and possible structural transformation of the nanosystems. As indicated in Figure 3, the samples retained their emission, while sample 1:0.25 presented the highest intensity. Samples 1:0.5 and 1:1 present a similar FS intensity. Also, a redshift in all peaks was observed (Table S5). Probably, the dye-loaded nanocarriers can reorganize after one week, resulting in the observed redshift.

**Figure 4:**
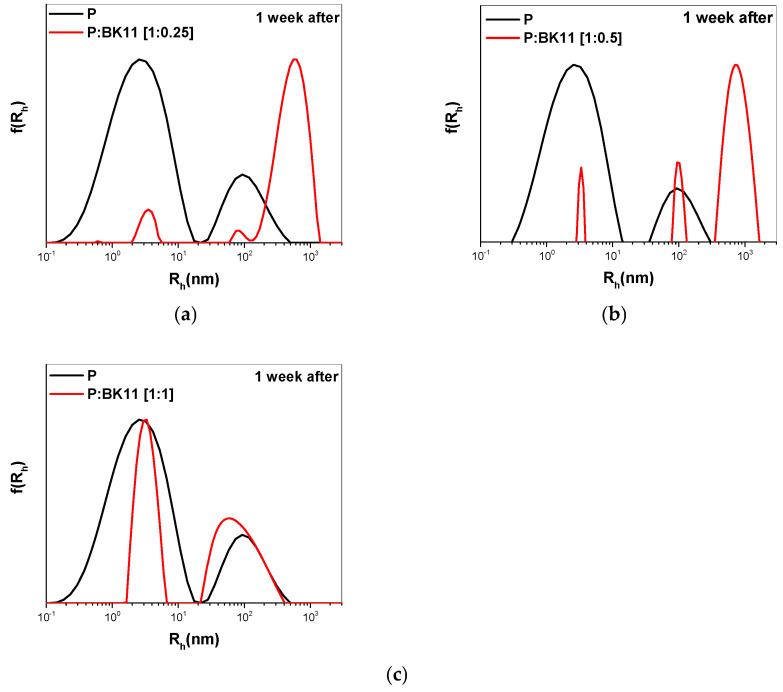
DLS measurements conducted after one week: polymer (black) and polymer–dye at ratios (**a**) 1:0.25 (red); (**b**) 1:0.5 (red); and (**c**) 1:1 (red).

DLS measurements performed after one week depicted the changes in the nanosystems. Free chains at 3 nm still exist, but in solutions of ratios 1:0.25 and 1:0.5, the particles became larger, and the scattered intensity was higher (Figure 4a–c). The sample at 1:1 presented smaller particles. The polydispersity index is also smaller for all cases (Table 2).

**Figure 5:**
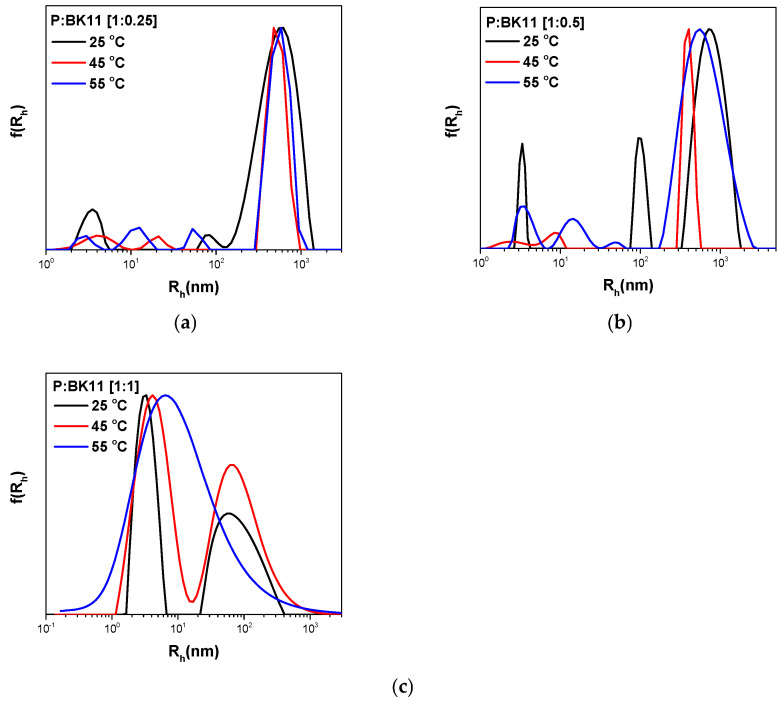
Temperature-dependent DLS measurements conducted after one week of nanosystem preparation: polymer–dye at ratios of (**a**) 1:0.25; (**b**) 1:0.5; and (**c**) 1:1.

As the polymer presents thermosensitivity, the solution of the co-assembled nanosystems was also measured at different temperatures (Figure 5). For the ratio of 1:0.25, there were no significant changes [30]. As far as the 1:0.5 ratio is concerned, a shift was observed toward a smaller radius. In the case of 1:1, as the temperature increased, the peaks became broader, and the radius increased. It seems that the presence of dye increases the thermoresponsive behavior of the polymer (Figure S9).

### 3.2. Co-Assembly of QP(DEGMA-co-DMAEMA-co-OEGMA) and BK11 Dye

**Figure 6:**
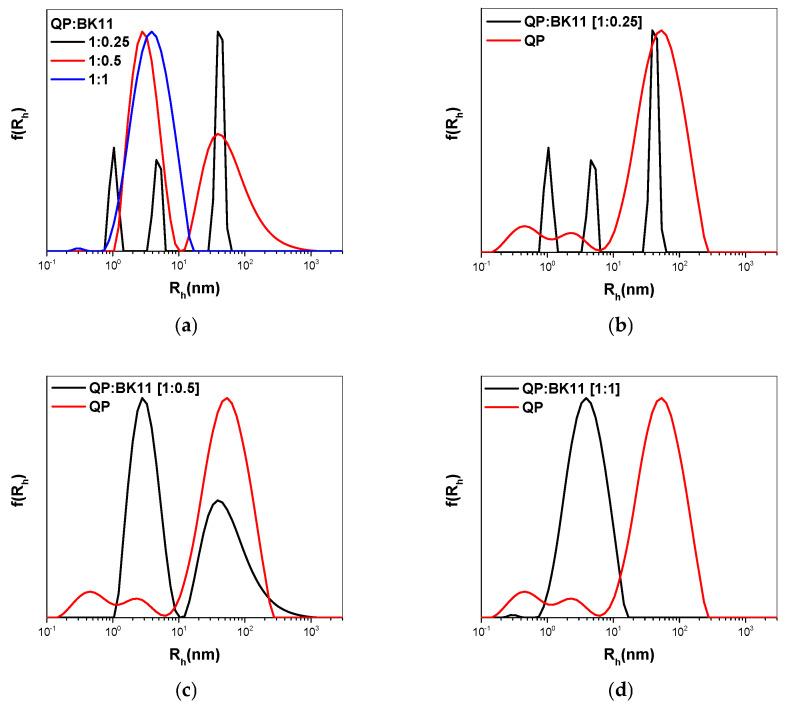
DLS measurements conducted on QP:BK11 nanosystems one day after preparation: (**a**) polymeric nanocarriers with loading ratios of 1:0.25 (black), 1:0.5 (red), and 1:1 (blue); (**b**) DLS measurements of polymer (red) and polymer–dye at ratio of 1:0.25 (black); (**c**) DLS measurements of polymer (red) and polymer–dye at ratio of 1:0.5 (black); and (**d**) DLS measurements of polymer (red) and polymer–dye at ratio of 1:1 (black).

In order to clarify the role of the tertiary amine groups in the electrostatic complexation of the polymer with the dye, the quaternization of the initial amino-functional polymer was performed. In Figure S10, the NMR measurement proved the successful quaternization of amine groups. The quaternized polymer was used for the co-assembly with BK11 AIE dye towards the formation of new nanocarriers. DLS measurements confirmed the formation of nanoparticles for both pure quaternized polymer and the dye-loaded nanocarriers (Table 3, Figure 6). ELS also proved the positive charge of the quaternized polymer and the decrease in charge of the nanoscale co-assemblies as the dye content was increased.

**Figure 7:**
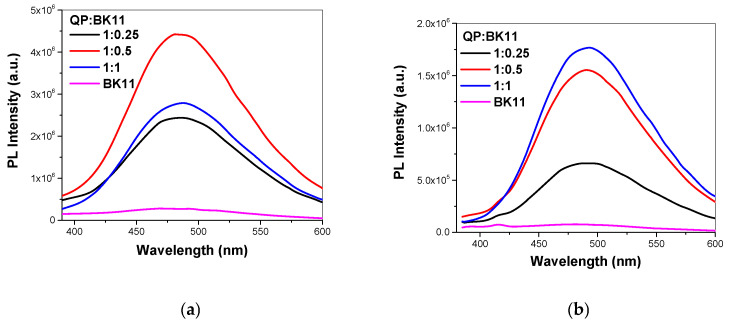
FS measurements conducted on dye (purple) and QP:BK11 co-assembled nanosystems with loading ratios of 1:0.25 (blue), 1:0.5 (red), and 1:1 (black) after one day of nanosystem formation: (**a**) excitation wavelength (317 nm) and (**b**) excitation wavelength (365 nm).

Observation of solutions under a UV lamp with the naked eye suggests that polymer quaternization resulted in stronger polymer/dye interactions compared to those of the pure dye. This indicates larger encapsulation and fluorescence ability of the dye (Figures S6 and S12). FS measurements proved that more dye was loaded into the nanoparticles and/or there is stronger complexation, which results in less freedom of dye movement/rotation since the fluorescence intensity was increased by one order of magnitude (Figure 7). Peaks appeared at 482 nm for the excitation wavelength at 317 nm, while it appeared at 493 for the 365 nm excitation (Tables S6 and S7). UV-Vis measurements confirmed the absorbance peaks of the dye. Increased absorbance was measured at the ratio of 1:1 (Figure S11).

**Figure 8:**
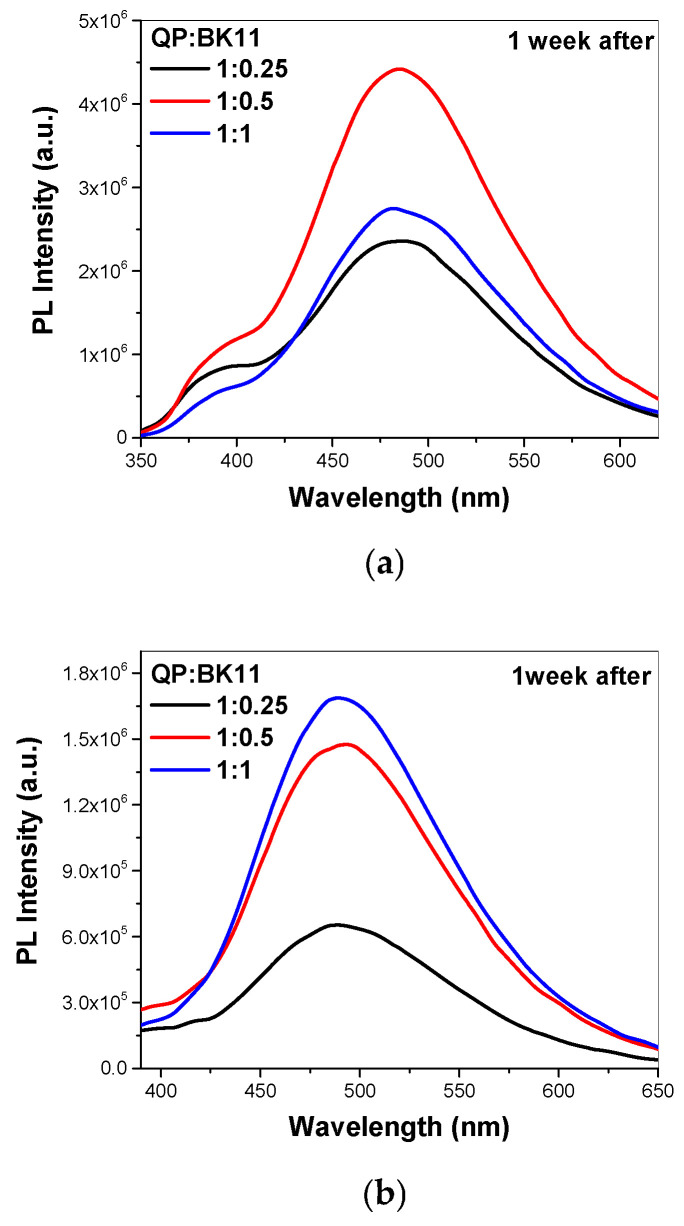
FS measurements conducted on QP:BK11 co-assembled nanosystems with loading ratios of 1:0.25 (black), 1:0.5 (red), and 1:1 (blue) one week after nanosystem formation: (**a**) excitation wavelength (317 nm) and (**b**) excitation wavelength (365 nm).

Measurements were repeated one week after the nanosystem preparation in order to observe the stability of the solutions. As it is depicted in Figure 8a,b, the systems changed, and the fluorescence increased in the 1:0.5 for the excitation wavelength of 317 nm. The polymer/dye ratio determines the conformation of the nanoaggregates and the fluorescence of the particles. In the case of 1:0.5, the maximum or the most efficient dye aggregation occurs, and for this reason, the FS intensity is higher. Furthermore, one more peak appeared, indicating that there is another type of aggregation taking place [32]. As for the excitation wavelength of 365 nm, the 1:1 ratio presents the maximum intensity. This can be attributed to the excitation of a different size or type of dye aggregate by this wavelength.

**Figure 9:**
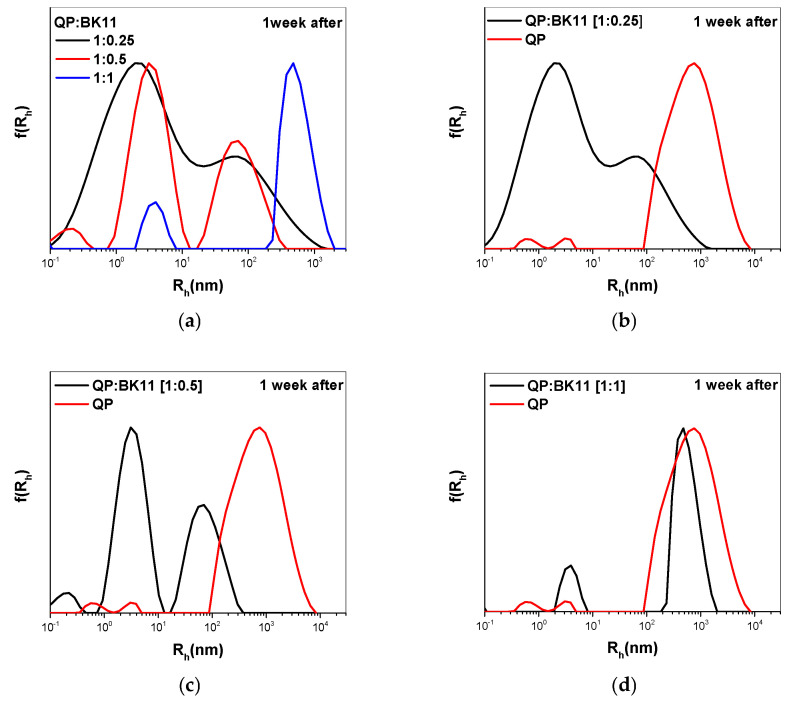
DLS measurements conducted on quaternized polymeric nanocarriers one week after preparation: (**a**) polymeric nanocarriers with loading ratios of 1:0.25 (black), 1:0.5 (red), 1:1 (blue); (**b**) DLS measurements of polymer (red) and polymer–dye at ratio of 1:0.25 (black); (**c**) DLS measurements of polymer (red) and polymer–dye at ratio of 1:0.5 (black); (**d**) DLS measurements of polymer (red) and polymer–dye at ratio of 1:1 (black).

DLS measurements were also taken one week after nanosystem preparation. A stronger scattered intensity and larger nanoparticles were observed in all systems indicating that there was increased aggregation in the solutions (Table 4, Figure 9).

### 3.3. FBS Interactions with Polymer–Dye Co-Assembled Nanoparticles

**Figure 10:**
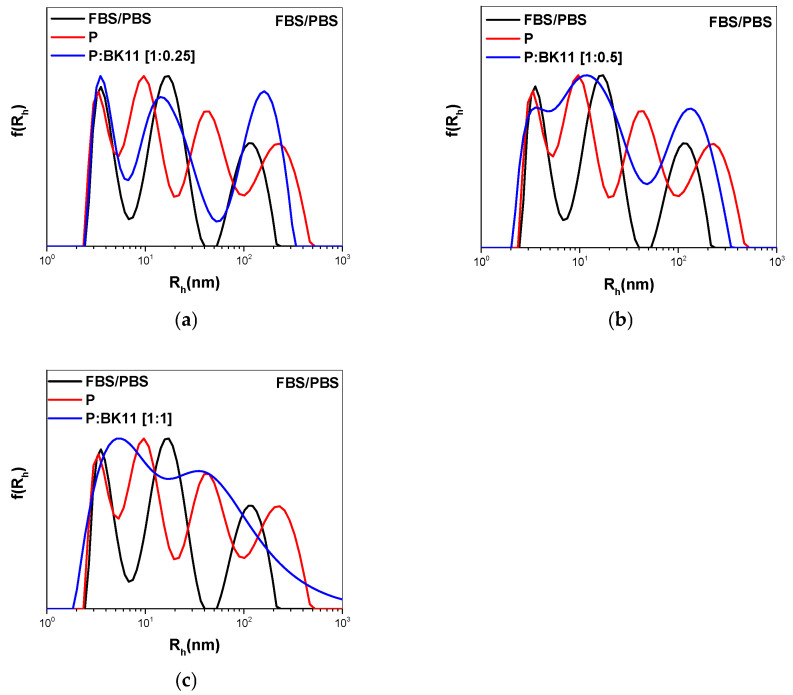
DLS measurements of polymer (red), FBS medium (black), and polymer–dye nanoparticles in FBS medium (blue): (**a**) ratio of 1:0.25; (**b**) ratio of 1:0.5; and (**c**) ratio of 1:1.

**Figure 11:**
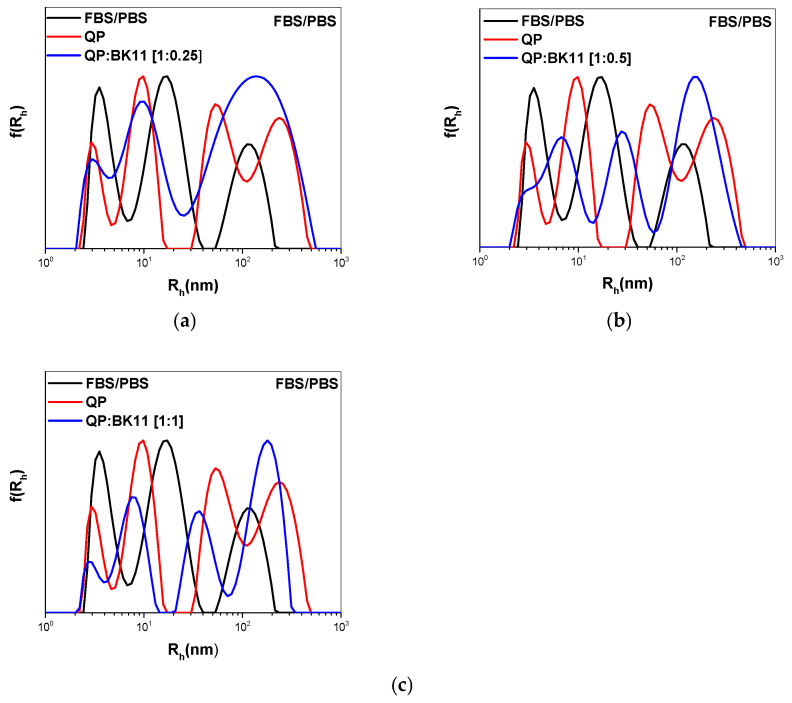
DLS measurements of quaternized polymer (red), FBS medium (black), and polymer–dye nanoparticles in FBS medium (blue): (**a**) ratio of 1:0.25; (**b**) ratio of 1:0.5; and (**c**) ratio of 1:1.

For the investigation of the interactions of the nanoparticles with human blood, DLS measurements were carried out in the FBS/PBS medium. The FBS/PBS characteristic peaks were observed in the pure biological solution of serum proteins [29]. Addition of the polymer in the medium results in a shift of all peaks to smaller dimensions, most probably denoting a decrease in the size of existing aggregates due to polymer/protein interactions. In the case of sample 1:0.25, the addition of dye resulted in a shift of peaks close to the peaks of FBS. For sample 1:1, a broad peak was observed covering all the FBS peaks (Figure 10). For the quaternized polymeric nanoparticles, the interactions were stronger in all cases as the polymer was positively charged, possibly forming aggregates with proteins in the FBS solution (Figure 11). Measurements were repeated after 72 h with no significant changes in the results (Figures S13 and S14). Only particles in the nanoscale region are observed in all cases.

### 3.4. NaCl Effects on Polymer–Dye Co-Assembled Nanoparticles

**Figure 12:**
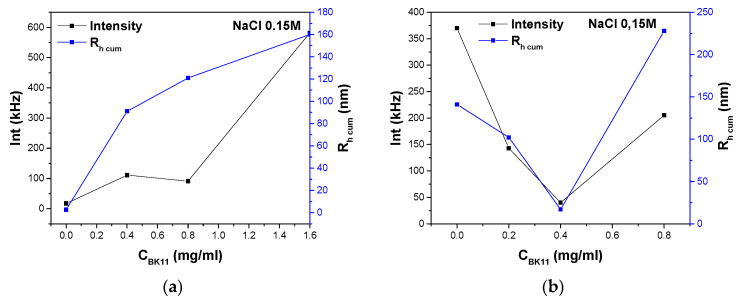
Effect of NaCl addition (at physiological levels) on co-assembled nanoparticles: (**a**) amino polymer precursor and (**b**) quaternized analog.

As discussed previously, electrostatic complexation occurs between the dye and the polymer. Addition of NaCl in the solution can affect the electrostatic interactions and may change the structure of the nanoaggregates. Therefore, the effect of NaCl on the size of the polymer–dye co-assembled particles at different dye concentrations was explored. For biological applications, the ionic strength of the aqueous media is substantial (in physiological conditions, the ionic strength is ca. 0.15 M NaCl), and the behavior of the nanoparticles (size, size distribution, possible secondary aggregation) under these ionic strength conditions should be tested as the first step [29]. As it is depicted in Figure 11, when the concentration of the dye increases, there is an increase in both the intensity and the radius of nanoparticles, indicating electrostatic interaction between the dye-loaded polymer particles and NaCl at salt concentration similar to physiological conditions. In the case of a quaternized polymer, a decrease is first observed up to a dye concentration of 0.4 mg/mLBK11, and then, an increase in intensity and radius is observed (Figure 12).

## 4. Conclusions

Herein, a terpolymer containing tertiary amine groups was utilized for the successful encapsulation of a sulphonated AIE dye. The experimental results proved the AIE phenomenon and the encapsulation of the dye inside the polymeric nanocarrier. The quaternization of the polymer showed an increase in dye emission as the polymer charge is crucial for the efficient electrostatic complexation and induces increased dye aggregation. Further research needs to be conducted in order to fully exploit the potential of the AIE phenomenon in nanomedicine. The present study paves the way for the creation of more biocompatible polymer–AIE hybrid systems for applications in bioimaging. Other future research directions would include pH or temperature-responsive polymeric nanocarriers and the encapsulation of drugs for applications in image-guided drug delivery.

## Figures and Tables

**Table 1:** DLS and ELS measurements conducted on P:BK11 nanoparticles.

Sample	Int _90_ (Kcps)	PDI	R_h, Cont_ (nm)	Zeta Potential (mV)
***p***	23	0.57	2.3	−0.4
			103	
**1:0.25**	108	0.57	3.3	−10.7
			283	
**1:0.5**	106	0.55	3.5	−1.8
			258	
**1:1**	87	0.6	3.6	−10.3
			245	

**Table 2:** DLS measurements conducted on P:BK11 carriers one week after formulation.

Sample	Int _90_ (Kcps)	PDI	R_h, Cont_ (nm)
**1:0.25**	235	0.5	3
			498
**1:0.5**	230	0.52	3 101
			758
**1:1**	37	0.48	3
			78

**Table 3:** DLS and ELS measurements conducted on QP:BK11 nanosystems one day after formation.

Sample	Int _90_ (Kcps)	PDI	R_h, Cont_ (nm)	Zeta Potential (mV)
**QP**	27	0.58	32	+60
**1:0.25**	21	0.56	1 5	+42
			42	
**1:0.5**	32	0.53	3	+29
			54	
**1:1**	29	0.39	4	−6.5

**Table 4:** DLS measurements of QP:BK11 nanocarriers conducted one week after formation.

Sample	Int _90_ (Kcps)	PDI	R_h, Cont_ (nm)
**QP**	130	0.52	1.3 685
**1:0.25**	15	0.56	2
			70
**1:0.5**	31	0.53	3 73
**1:1**	183	0.56	4
			550

## Data Availability

Dataset available on request from the authors.

## Supplementary Materials

The following supporting information can be downloaded at: https://www.mdpi.com/article/10.3390/nano14201631/s1, Scheme S1. Synthesis of sodium tetraphenylethylene 4,4′,4″,4‴-tetrasulfonate (ΒΚ11); Figure S1. ^1^H-NMR (250 MHz, CDCl_3_, up) and ^13^C-NMR (63 MHz, CDCl_3_, down) spectra of TPE; Figure S2. ^1^H-NMR (250 MHz, D_2_O, up) and ^13^C-NMR (63 MHz, D_2_O, down) spectra of TPE-SA; Figure S3. ^1^H-NMR (250 MHz, D_2_O, up) and ^13^C-NMR (63 MHz, D_2_O, down) spectra of BK11; Figure S4. UV-Vis spectrum of BK11 dye; Table S1. Fluorescence data for BK11 dye in water, λ_exc_ = 317 nm; Table S2. Fluorescence data, λ_exc_ = 365 nm; Figure S5. Fluorescence spectra of BK11 dye; Figure S6. BK11 dye aqueous solution under UV lamp, λ = 365 nm; Figure S7: Size distribution from DLS measurement for BK11 dye in water; Table S3. DLS data for BK11 dye aqueous solution; Figure S8. UV-Vis spectra of dye-loaded polymeric nanocarriers; Table S4. Fluorescence measurements conducted one day after formation of polymer–dye nanoparticles, λ_exc_ = 365 nm; Table S5. Fluorescence measurements conducted one week after formulation, λ_exc_ = 365 nm; Figure S9. Temperature-dependent DLS measurements conducted after one week of preparation: (a) amino polymer; (b) polymer–dye at ratio of 1:0.25; (c) polymer–dye at ratio of 1:0.5; and (d) polymer–dye at ratio of 1:1; Figure S10. ^1^H-NMR spectrum of quaternized polymer; Figure S11. UV-Vis measurement of the quaternized polymeric nanocarriers loaded with dye; Table S6. Fluorescence measurements conducted one day after formulation (quaternized polymer), λ_exc_ = 317 nm; Table S7. Fluorescence measurements conducted one day after formulation (quaternized polymer), λ_exc_ = 365 nm; Figure S12. Quaternized polymer–dye co-assembled nanoparticles under UV lamp, λ_exc_ = 365 nm; Table S8. Fluorescence measurements conducted one week after formulation, λ_exc_ = 317 nm; Table S9. Fluorescence measurements conducted one week after formulation, λ_exc_ = 365 nm; Figure S13. DLS size distributions of FBS nanoparticle solutions after 72 h: (a) polymer–dye at ratio of 1:0.25; (b) polymer–dye at ratio of 1:0.5; and (c) polymer-dye at ratio of 1:1; Figure S14. DLS size distributions of FBS nanoparticle solutions after 72 h: (a) quaternized polymer–dye at ratio of 1:0.25; (b) quaternized polymer–dye at ratio of 1:0.5; and (c) quaternized polymer–dye at ratio of 1:1. Refs. [33,34] are cited in the Supplementary Materials document.
