# Supplementary material for: Co-Assembled Nanosystems Exhibiting Intrinsic Fluorescence by Complexation of Amino Terpolymer and Its Quaternized Analog with Aggregation-Induced Emission (AIE) Dye
Source: Nanomaterials (Basel). 2024 Oct 11;14(20):1631. doi: 10.3390/nano14201631 (PMC11510664; doi:10.3390/nano14201631)
Supplement: Supplementary file 1 [file nanomaterials-14-01631-s001.zip › nanomaterials-3206643-supplementary.pdf]

Supplementary Materials

Co-Assembled Nanosystems Exhibiting Intrinsic Fluorescence by Complexation of Amino Terpolymer and Its Quaternized Analog with Aggregation-Induced Emission (AIE) Dye

Michaila Akathi Pantelaiou [1], Dimitrios Vagenas [1], Evangelos S. Karvelis [2], Georgios Rotas [2] and Stergios Pispas [1,*]

[1] Theoretical and Physical Chemistry Institute, National Hellenic Research Foundation, 48 Vassileos Constantinou Ave., 11635 Athens, Greece; akathi39@gmail.com (M.A.P.), dimitrisv98@gmail.com (D.V.)
[2] Section of Organic Chemistry and Biochemistry, Department of Chemistry, University of Ioannina, 45110 Ioannina, Greece; ch05879@uoi.gr (E.S.K.), rotasgiorgos@uoi.gr (G.R.)
* Correspondence: pispas@eie.gr

Synthesis of BK11 AIE dye

Reagents were purchased from commercial suppliers and were used without further purification. Tetrahydrofuran was distilled dry from sodium/benzophenone. Argon gas was used for inert atmosphere. NMR spectra were recorded on a Bruker Avance 250 MHz instrument.

Scheme S1. Synthesis of sodium tetraphenylethylene 4,4',4'',4'''-tetrasulfonate (**BK11**)

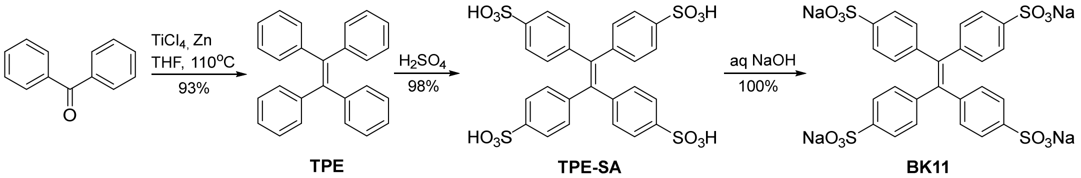

Synthesis of tetraphenylethylene (**TPE**) [33]

Zinc dust (0.63 g, 9.875 mmol) was added in a flame dried pressure tube with septum under Ar and flame dried again. Dry THF (3 mL) was added, followed by dropwise addition of $TiCl_4$ (0.35 mL, 3.3 mmol) in the stirring mixture. The tube was capped and stirred at 110°C for 1h. Then brought to r.t., cap was replaced with septum/Ar, benzophenone (0.5 g, 2.75 mmol) was added, tube capped again and stirred at 110°C for another 1h. Then brought to r.t., the reaction mixture

was quenched with 1M NH_4Cl (10 mL), water (10 mL) was added, and the mixture was carefully washed with dichloromethane (4x20 mL), avoiding emulsion. The combined organic phases were dried (Na_2SO_4) and solvent evaporated. Recrystallization of the residue from isopropyl alcohol afforded **TPE**[1] as colorless crystals (425 mg, 93%). ^{1}H-NMR (250 MHz, $CDCl_3$)δ: 7.13 – 7.11 (m, 12H), 7.09 – 7.04 (m, 8H). ^{13}C-NMR (63 MHz, $CDCl_3$) δ: 143.86, 141.10, 131.46, 127.78, 126.54 ppm.

Synthesis of tetraphenylethylene 4,4',4'',4'''-tetrasulfonic acid (**TPE-SA**) [34]

TPE (0.25 g, 0.75 mmol) was suspended in H_2SO_4 (97%, 5 ml, 93.3 mmol) and the mixture was stirred for 3h at 110°C, the oil bath was removed and stirred for another 1h. The resulting solution was decanted onto stirring ice cold water (2.5 mL), then ethyl acetate (5 mL) was added, the resulting precipitate was filtered and washed with ethyl acetate (2x2 mL), leaving **TPE-SA** as white solid (480 mg, 98%). ^{1}H-NMR (250 MHz, D_2O) δ: 7.55 (d, *J* = 8.3 Hz, 8H), 7.23 (d, *J* = 8.3 Hz, 8H). ^{13}C-NMR (63 MHz, $CDCl_3$) δ: 145.43, 141.04, 140.91, 131.61, 125.07 ppm.

Synthesis of sodium tetraphenylethylene 4,4',4'',4'''-tetrasulfonate (**BK11**) [34]

Sodium hydroxide (0.06 g, 1.53 mmol) was added in a solution of **TPE-SA** (250mg, 0.38mmol) in distilled water (5.4 mL), and the whole was stirred for 1h. Then, the water was removed was removed via lyophilization, leaving **BK11** as yellow solid (281 mg, 100%). ^{1}H-NMR (250 MHz, D_2O) δ: 7.61 (d, *J* = 8.5 Hz, 8H), 7.29 (d, *J* = 8.4 Hz, 8H).^{13}C-NMR (63 MHz, $CDCl_3$) δ: 145.47, 141.09, 140.96, 131.66, 125.13 ppm.

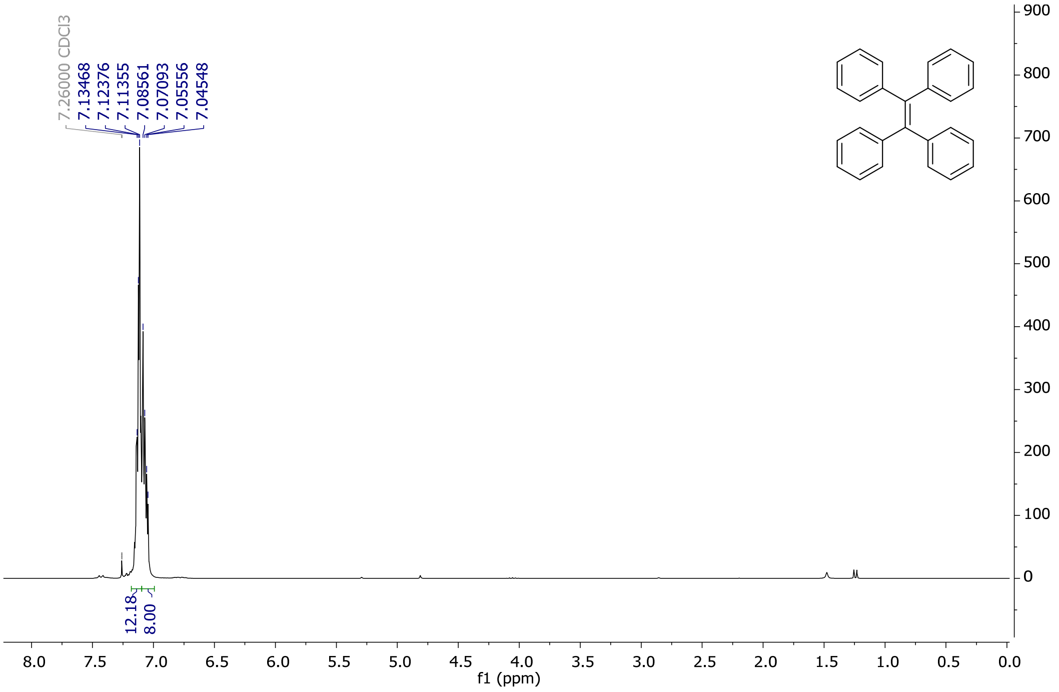

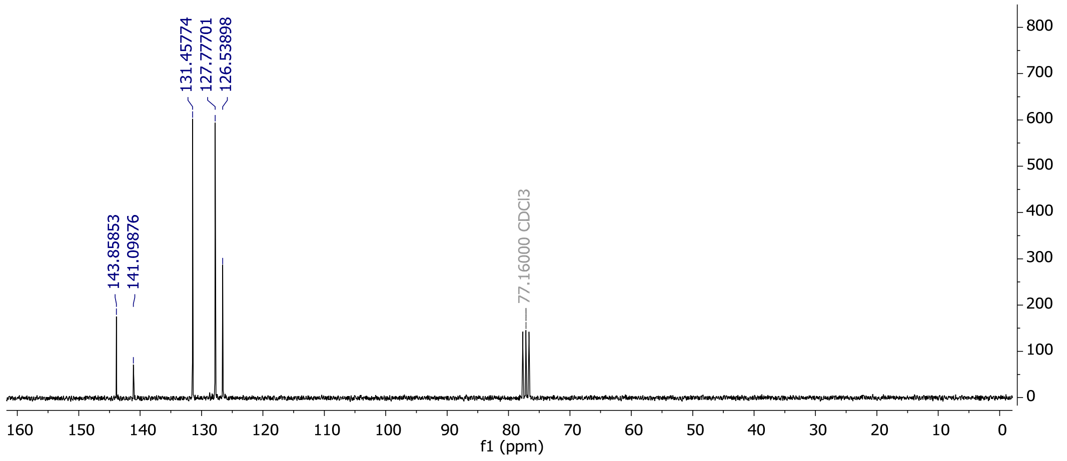

Figure S1. ^{1}H-NMR (250 MHz, $CDCl_3$, up) and ^{13}C-NMR (63 MHz, $CDCl_3$, down) spectra of **TPE**.

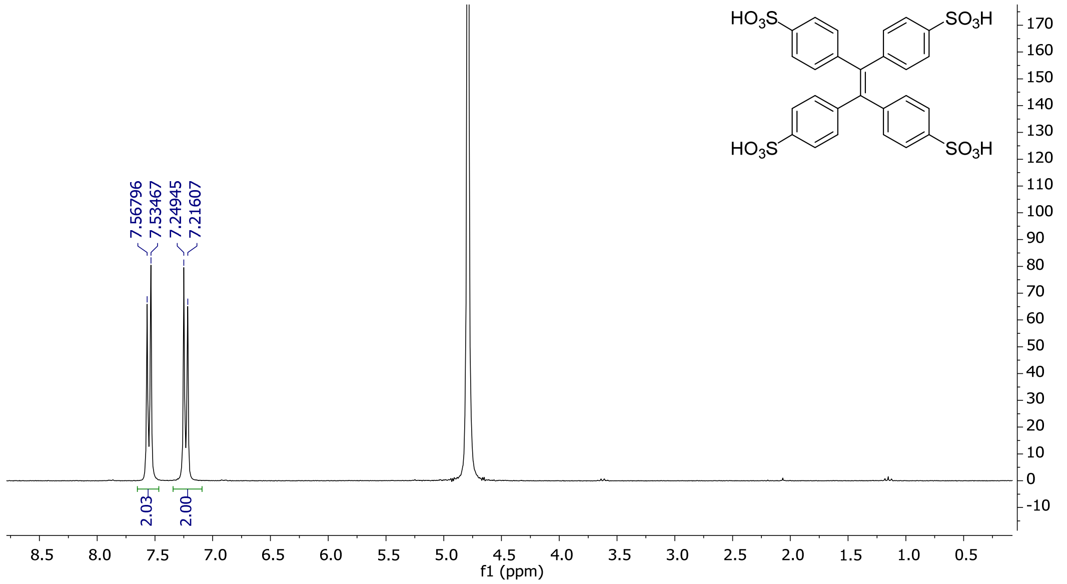

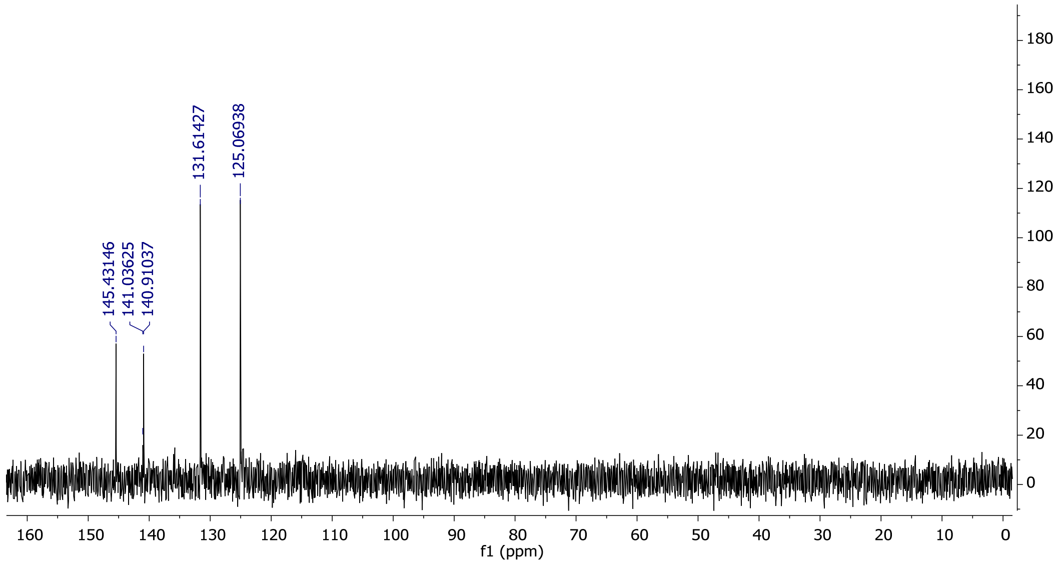

Figure S2. ^{1}H-NMR (250 MHz, D_2O, up) and ^{13}C-NMR (63 MHz, D_2O, down) spectra of **TPE-SA**.

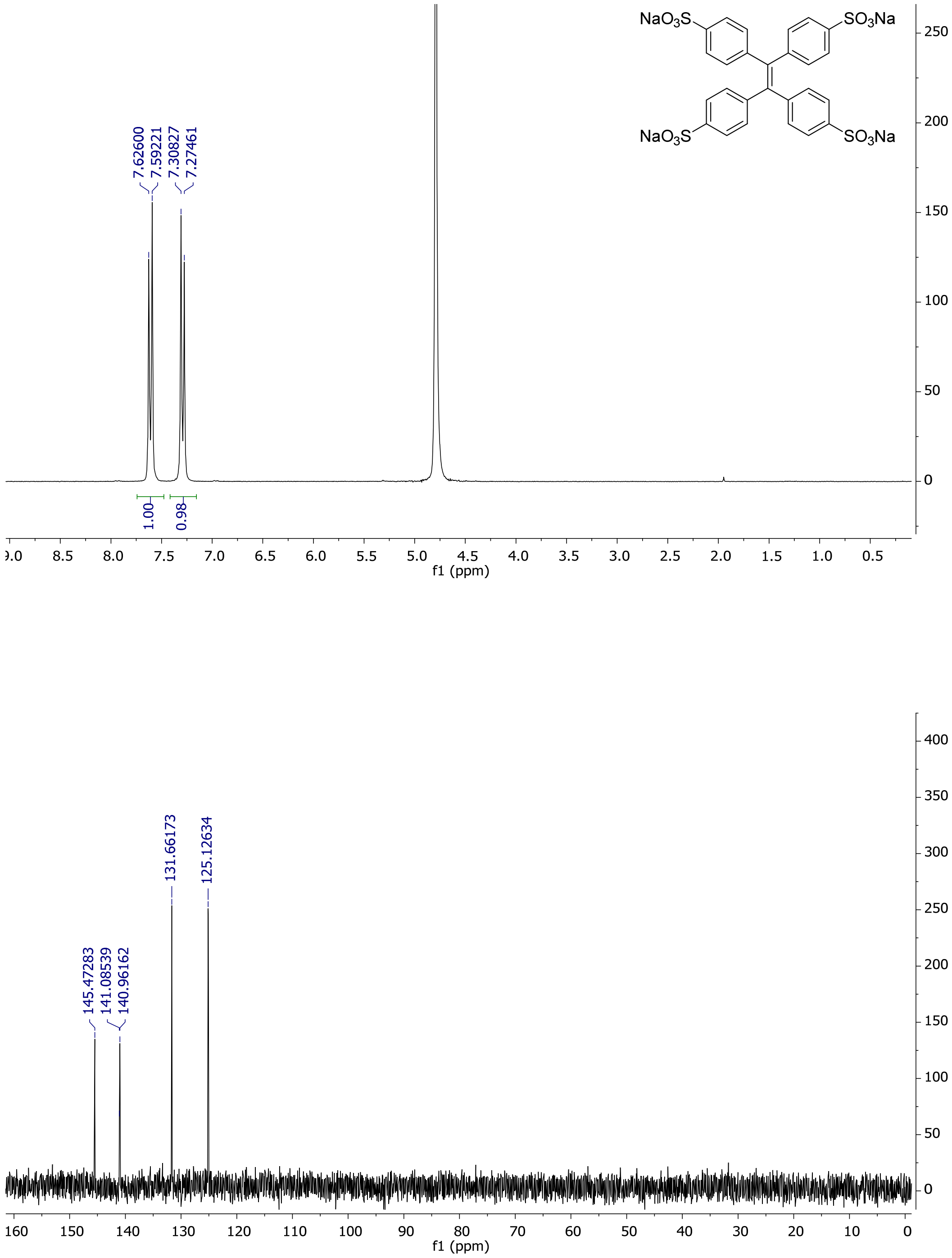

Figure S3. ^{1}H-NMR (250 MHz, D_2O, up) and ^{13}C-NMR (63 MHz, D_2O, down) spectra of **BK11**.

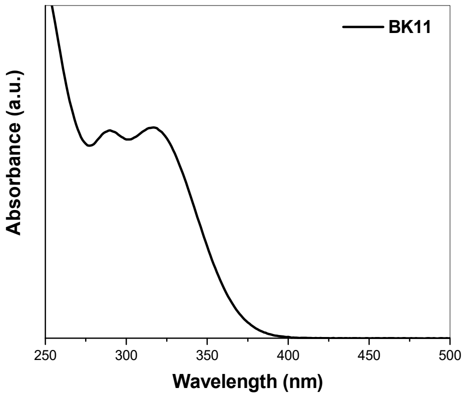

Figure S4.UV-Vis spectrum of BK11dye.

Table S1. Fluorescence data for BK11 dye in water, λ_{exc}= 317nm.

Sample	λ_{max} (nm)	Intensity (a.u.)
**BK11**	471	2.8 x10^5
	491	2.76 x10^5

Table S2. Fluorescence data, λ_{exc}= 365nm.

Sample	λ_{max} (nm)	Intensity (a.u.)
**BK11**	415	7.43 x10^4
	480	7.78 x10^4

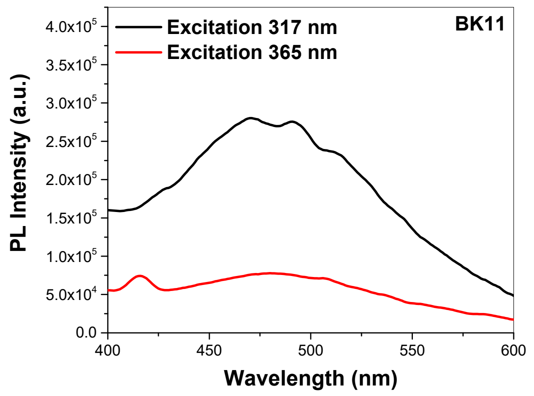

Figure S5. Fluorescence spectra of BK11 dye.

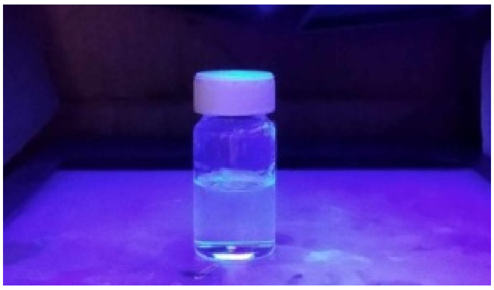

Figure S6. BK11 dye aqueous solution under UV lamp, λ=365nm.

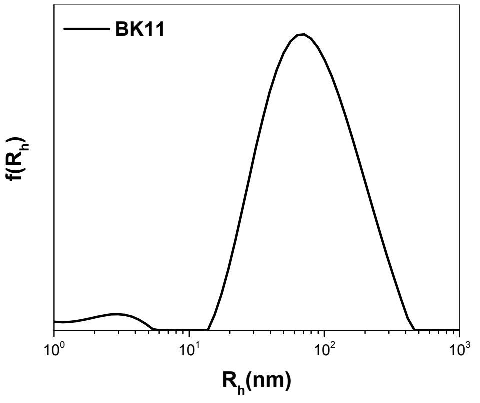

Figure S7. Size distribution from DLS measurement for BK11 dye in water.

Table S3. DLS data for BK11 dye aqueous solution.

**Sample**	**Int $_{90}$ (Kcps)**	**PDI**	**$R_{h, Cont}$ (nm)**
**BK11**	23	0.54	96

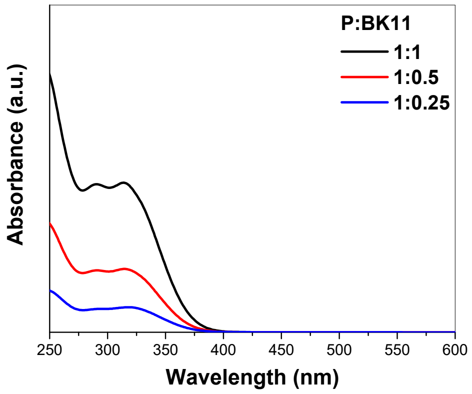

Figure S8. UV-Vis spectra of dye loaded polymeric nanocarriers.

Table S4. Fluorescence measurements one day after formation of polymer-dye nanoparticles, λ_{exc}= 365nm.

Sample	λ_{max} (nm)	Intensity (a.u.)
**1:0.25**	488	1.19×10^6
**1:0.5**	479	1.26×10^6
**1:1**	491	9.6×10^5

Table S5. Fluorescence measurements one week after formulation, λ_{exc}= 365nm.

Sample	λ_{max} (nm)	Intensity (a.u.)
**1:0.25**	490	1.66×10^6
**1:0.5**	493	1.44×10^6
**1:1**	491	1.45×10^6

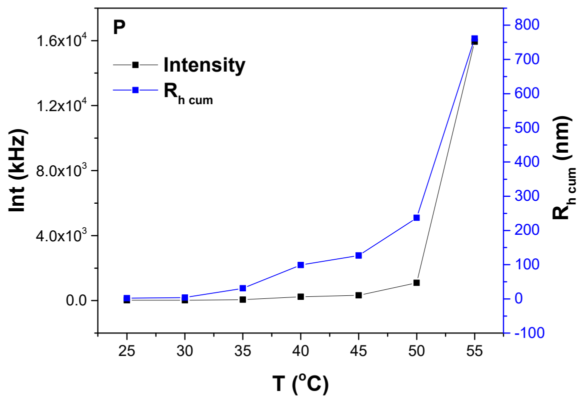

(a)

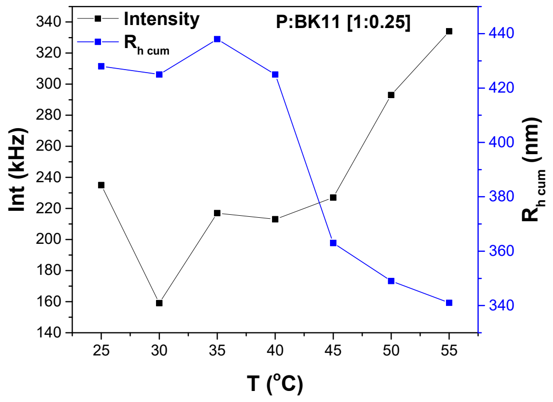

(b)

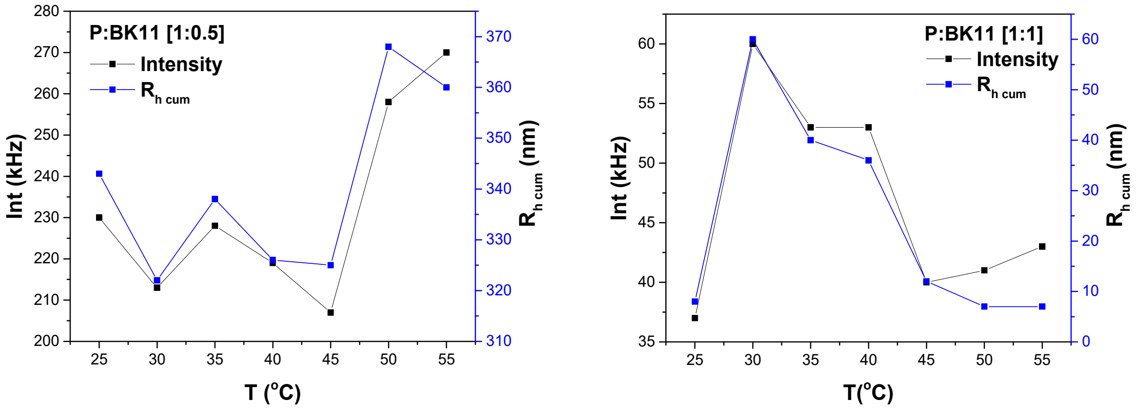

(c) (d)

Figure S9. Temperature dependent DLS measurements after one week of preparation: (**a**) Amino Polymer; (**b**) Polymer-dye at ratio 1:0.25; (**c**) Polymer-dye at ratio 1:0.5; (**d**) Polymer-dye at ratio 1:1.

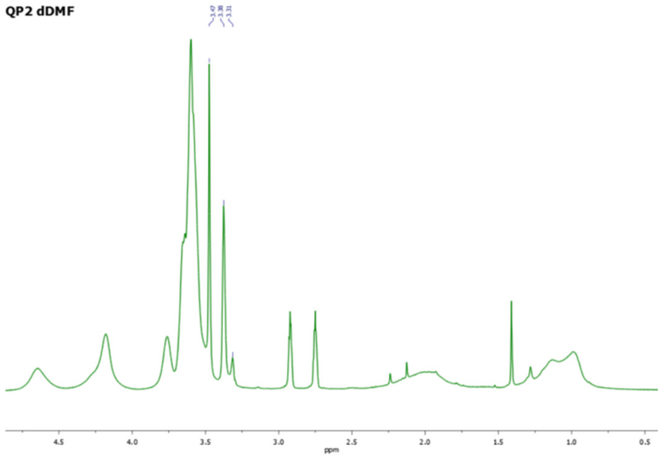

Figure S10. ^{1}H-NMR spectrum of quaternized polymer.

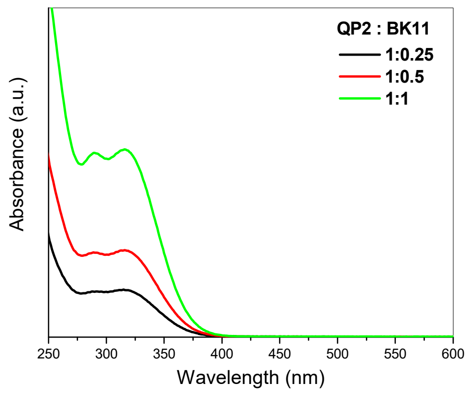

Figure S11. UV-Vis measurement of the quaternized polymeric nanocarriers loaded with dye.

Table S6. Fluorescence measurements one day after formulation (quaternized polymer), λ_{exc}= 317nm.

Sample	λ_{max} (nm)	Intensity (a.u.)
**1:0.25**	486	2.44x10^6
**1:0.5**	482	4.43x10^6
**1:1**	488	2.79x10^5

Table S7. Fluorescence measurements one day after formulation (quaternized polymer), λ_{exc}= 365nm.

Sample	λ_{max} (nm)	Intensity (a.u.)
**1:0.25**	495	6.6x10^5
**1:0.5**	491	1.55x10^6
**1:1**	493	1.77x10^6

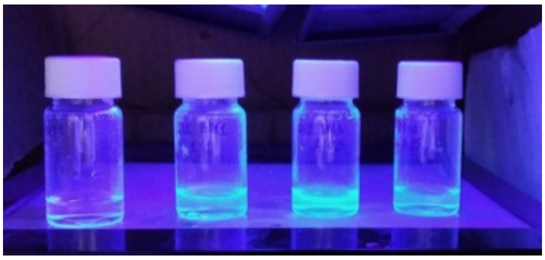

Figure S12. Quaternized polymer-dye co-assembled nanoparticles under UV lamp, λ_{exc}= 365 nm. From left to right BK11 dye solution, 1:0.25, 1:0.5 and 1:1 mixtures on day one (as of Table S6).

Table S8. Fluorescence measurements one week after formulation, λ_{exc}= 317nm.

Sample	λ_{max} (nm)	Intensity (a.u.)
**1:0.25**	398	$8.6x10^5$
	485	$2.36x10^6$
**1:0.5**	398	$1.17x10^6$
	485	$4.42x10^6$
**1:1**	397	$6x10^5$
	482	$2.75x10^6$

Table S9. Fluorescence measurements one week after formulation, λ_{exc}= 365nm.

Sample	λ_{max} (nm)	Intensity (a.u.)
**1:0.25**	490	$6.53x10^5$
**1:0.5**	492	$1.48x10^6$
**1:1**	489	$1.69x10^6$

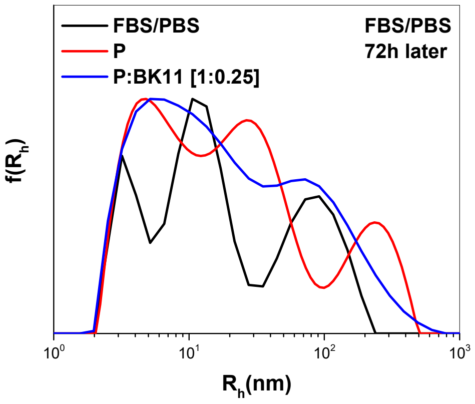

(a)

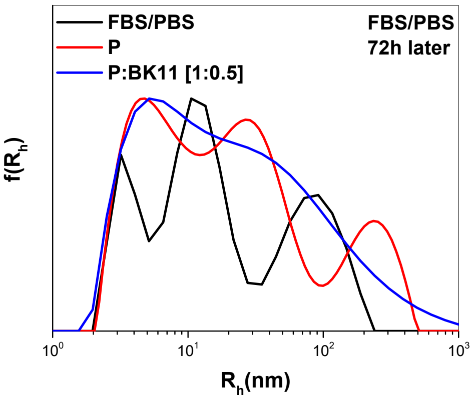

(b)

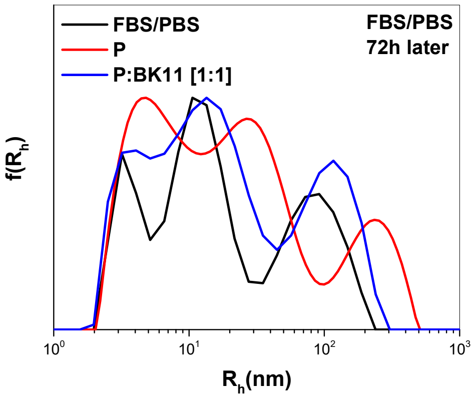

(c)

Figure S13. DLS size distributions of FBS nanoparticle solutions after 72 hours: (**a**) Polymer-dye at ratio 1:0.25; (**b**) Polymer-dye at ratio 1:0.5; (**c**) Polymer-dye at ratio 1:1.

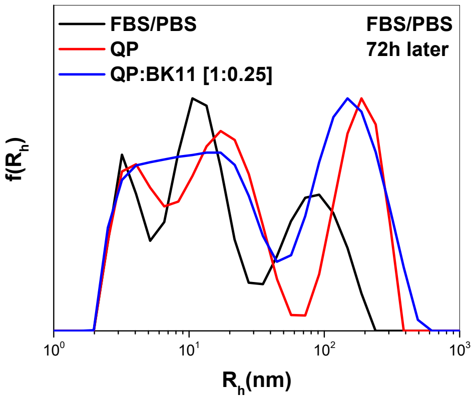

(a)

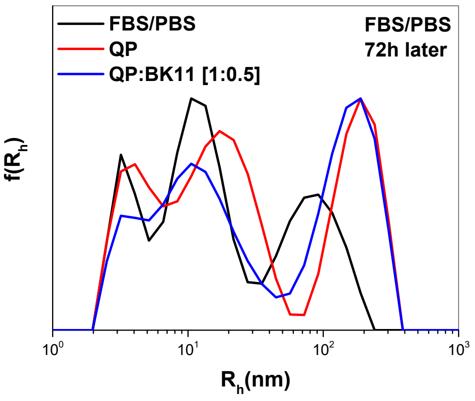

(b)

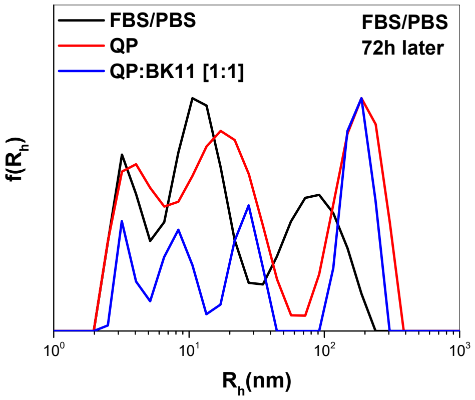

(c)

Figure S14. DLS size distributions of FBS nanoparticle solutions after 72 hours: (**a**) Quaternized polymer-dye at ratio 1:0.25; (**b**) Quaternized polymer-dye at ratio 1:0.5; and (**c**) Quaternized polymer-dye at ratio 1:1.

References

33. Mohamed, M.G.; Ahmed, M.M.M.; Du, W.-T.; Kuo, S.-W. Meso/Microporous Carbons from Conjugated Hyper-Crosslinked Polymers Based on Tetraphenylethene for High-Performance CO_2 Capture and Supercapacitor. *Molecules* **2021**, *26*, 738. https://doi.org/10.3390/molecules26030738

34. Zhao, Y.-K.; Gao, Z.-Z.; Wang, H.; Zhang, D.-W.; Li, Z.-T. Self-Assembly of Supramolecular Polymers in Water from Tetracationic and Tetraanionic Monomers in Water through Cooperative Electrostatic Attraction and Aromatic Stacking. *Chin*. *Chem*. *Lett*. **2019**, *30*, 127. https://doi.org/10.1016/j.cclet.2018.10.016
